# Nurses’ perspectives on professional self-concept and its influencing factors: A qualitative study

**DOI:** 10.1186/s12912-024-01834-y

**Published:** 2024-04-09

**Authors:** Chuyuan Miao, Chunqin Liu, Ying Zhou, Xiaofang Zou, Liqin Song, Joanne W.Y. Chung, Wenying Tan, Xiaohua Li, Dong Li

**Affiliations:** 1 School of Nursing, Guangzhou Medical University, Guangzhou, Guangdong Province 510182 China; 2 Department of Nursing, The Third Affiliated Hospital of Guangzhou Medical University, Guangzhou, Guangdong Province 510150 China; 3 Kiang Wu Nursing College of Macau, Macao, Macao SAR 999078 China; 4 Department of International Culture Education, Chodang University, Muan, 58530 Republic of Korea

**Keywords:** Nurses, Professional self-concept, Qualitative study, Factors

## Abstract

**Background:**

Nurses with a strong professional self-concept tend to exhibit a positive mindset and strong work engagement, delivering high-quality patient care. Although numerous quantitative studies have examined the factors impacting professional self-concept, there remains a limited exploration of these factors from the perspective of nurses themselves.

**Methods:**

This qualitative descriptive study uses the PERMA theory and Social Cognitive Theory as the theoretical framework. Semi-structured interviews were conducted with 15 nurses from six public hospitals in China. The data were analyzed thematically using a combination of inductive and deductive approaches.

**Results:**

Nurses’ understanding of professional self-concept could be divided into four categories: professional identity, competence, care, and knowledge. Factors influencing nurses’ professional self-concept were categorized into eight subthemes in three domains: (1) personal factors, including psychological qualities and attitude towards the nursing profession; (2) occupational-related behavioral factors, including role-oriented behavior and knowledge-oriented behavior; and (3) work environment and external factors, including external evaluation and perceptions of nurses, time allocation, nursing work tasks, work atmosphere, school education, and perceived supports.

**Conclusions:**

This study found that, although nurses had different personal experiences, their perceptions of professional self-concept were similar. Nurses’ professional self-concept is a multidimensional concept and involves various factors, such as personality, work-related characteristics, environment, and family. To thrive in a nursing career, nurses must discern the factors that can enhance or hinder their professional self-concept. By identifying and adjusting these factors, personalized support and positive interventions can be tailored to meet nurses’ specific needs, which ultimately nurtures their professional development.

**Trial registration:**

This study was registered on December 14, 2022, in the Chinese Clinical Trial Registry (ChiCTR2200066699) as part of our ongoing study.

**Supplementary Information:**

The online version contains supplementary material available at 10.1186/s12912-024-01834-y.

## Background

Nurses’ professional self-concept reflects their attitudes, perceptions, emotions, ethics, behaviors, and values in the nursing profession [1, 2]. Nurses are indispensable members of the healthcare team and assume the important responsibilities of caring for patients, providing medical services, and ensuring patient safety. Nurses’ professional development is a lifeline for the quality of hospital nursing services, which is related to the stability of the nursing team. Professional self-concept is the core of self-understanding of the profession and personal career development [2]; thus, it has long been receiving attention [3, 4].

Studies have shown that nurses’ professional self-concept is closely related to their mental health and that nurses with higher self-concept tend to maintain good job flexibility and strive to overcome difficulties encountered at work; therefore, they experience higher job satisfaction, motivation and retention intention [5–7]. Goliroshan et al. found that professional self-concept could significantly predict burnout among clinical nurses [8]. This implies that enhancing professional self-concept may mitigate burnout. People whose career matches their self-concept perceive their careers as meaningful and rewarding activities. Nurses in this position have a more favorable professional image and provide superior patient care. Conversely, nurses with a negative self-concept often feel disappointed with their abilities, lack motivation in their work, and perceive nursing as an unsatisfying and sad profession. As such, developing a professional self-concept is of great importance to nurses, particularly in terms of their career satisfaction and willingness to stay in the profession.

Furthermore, owing to the enormous contribution of nurses worldwide during the COVID-19 pandemic, more attention has been paid to them. During this public crisis, nurses explored their self-roles and remained in their professional roles while they defended their own health and avoided the risk of infection, leading them to have a deeper understanding of their roles and professional missions [9, 10]. Hence, exploring nurses’ understanding of their professional self-concept, especially after the COVID-19 pandemic, can provide a comprehensive understanding of how nurses perceive their competencies and values and identify suggestions to help them develop in their careers [11].

Existing research on nurses’ professional self-concept is primarily quantitative [4, 12]. It is reported that a high level of professional self-concept among new or junior nurses affects their willingness to stay and their career plans [6, 13]. Conversely, a high level of professional self-concept among senior nurses, such as nurse managers, affects their level of positive decision-making, as they take on more leadership responsibilities and are central to the development of the nursing team [14]. However, further qualitative research with a unique perspective is required to gain a deeper understanding of this content and to explore its influencing factors. In addition, previous research on the influencing factors and opinions related to the professional self-concept of nurses has suggested that professional self-concept may be differently influenced by public images, work environments, and cultural backgrounds [15–17]. Needless to say, a deeper exploration to investigate the factors that may influence nurses’ professional self-concept from their own perspective is essential. To sum up, this study explored nurses’ perceptions of their professional self-concept and its influencing factors using a qualitative research approach. This study aimed to promote nurses’ professional development, create high-quality nursing services, and help to formulate related training and intervention strategies in the future.

## Theoretical background

This study adopted the PERMA theory and Social Cognitive Theory (SCT) to explore nurses’ perspectives on professional self-concept and its influencing factors. This study sought to enable future nursing managers to provide targeted interventions from a psychological perspective.

Positive psychology advocates a positive perspective on individual well-being and focuses on human emotions, qualities, happiness, meaning, and fulfillment to pursue a good and happy life [18]. Professional self-concept is closely related to an individual’s career development and well-being [18, 19], and nurses’ professional self-concept can be considered an important reflection of their pursuit of a meaningful and happy life. Seligman et al. [18] proposed the PERMA theory on positive psychology, in which well-being refers to a flourishing life and consists of five main elements: Positive Emotion (P), such as pleasantness, joy, and other subjective feelings; Engagement (E) which refers to complete concentration and immersion in something; Relationships (R), which refers to the emotional (e.g., co-operation or exclusion) and behavioral (e.g., proximity or estrangement) aspects that arise in the course of an individual’s getting along with other people; Meaning (M), which refers to an individual’s pursuit of a certain sense of value and happiness in their life; and Accomplishment (A), which refers to an individual’s feeling of pleasure or success after accomplishing something. Similarly, social psychology stresses the pursuit of a meaningful life. According to Bandura, an individual’s perceived well-being comes from a level that matches their life goals; thus, the SCT pursues well-being from the perspective of individual values [20]. From this perspective, the SCT can also be combined with the PERMA theory. For instance, positive emotion may develop in interactions with others [21, 22]; For the engagement element, individuals may be influenced by others to develop a great interest, enthusiasm, and motivation for learning, enhancing the sense of engagement in themselves. Individuals may maintain interpersonal relationships by establishing good social behaviors with others; in other words, individuals are influenced by social roles to find meaning in life and work [23]; Regarding the accomplishment element, individuals may be influenced by others to set their own goals and self-development [22]. Therefore, this study combined the PERMA theory and SCT using the above five elements to design an interview outline and analyze nurses’ perceptions of their professional self-concept.

Furthermore, the interpretation of professional self-concept requires individual perception, which may be influenced by the individual’s own understanding, work, and life experiences, as well as other factors such as society, culture, race, religion, organization, and profession [24, 25]. In other words, the formation of professional self-concept results from the interactions between humans and environmental factors. Therefore, the influence of social factors should also be considered. According to Bandura, human activity is determined by the interaction between three factors: personal factors (i.e., individual cognition), behavioral factors, and the external environment [18, 26]. As a part of social psychology theory, the SCT stresses the significance of motivation and meaningful life, focusing on the self [18, 27], which further guides us in categorizing the factors that affect professional self-concept.

**Fig. 1:**
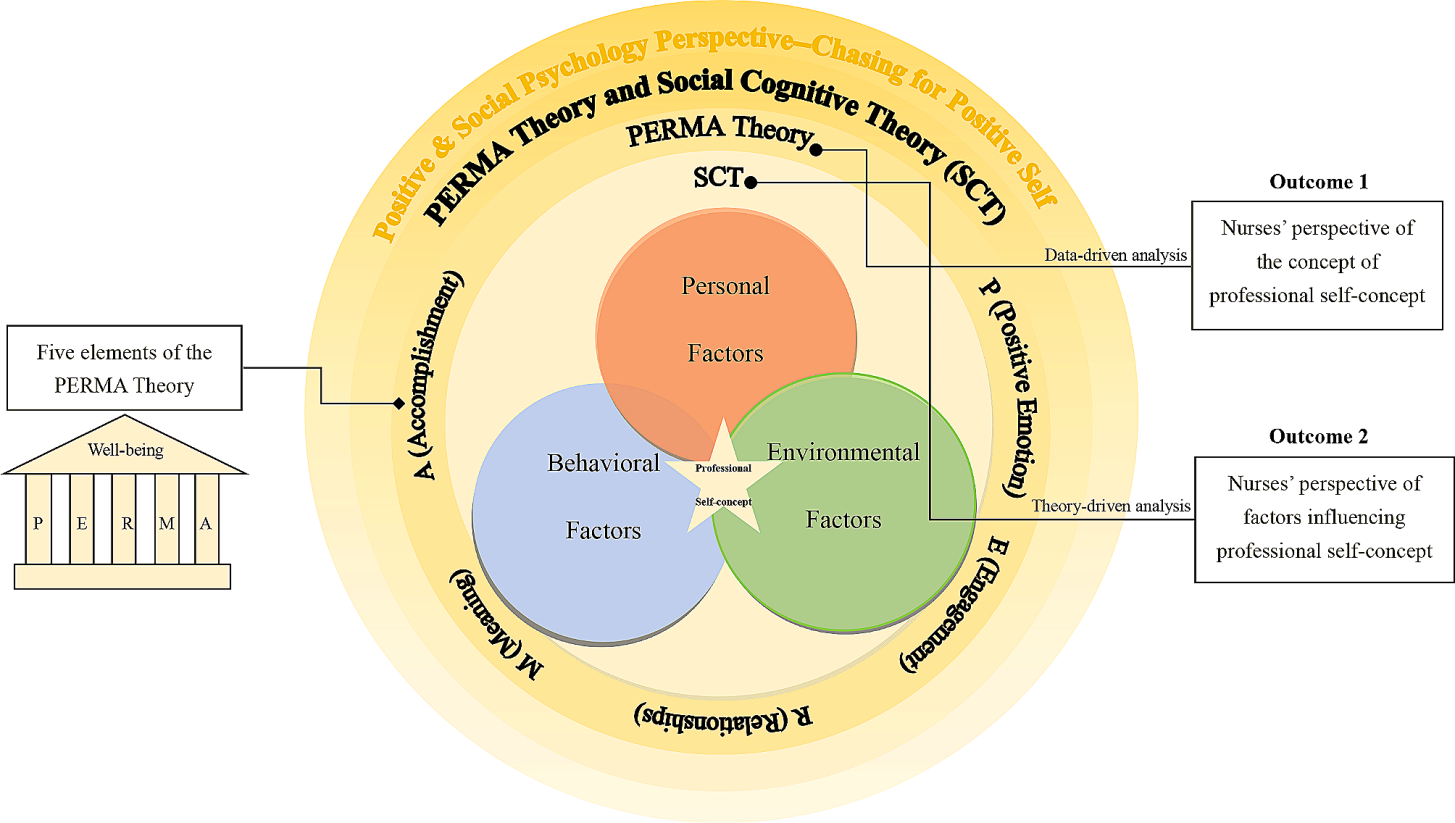
The theoretical framework of nurses’ professional self-concept and its influencing factors

The theoretical framework of this study is illustrated in Fig. 1. The middle circle introduces Martin Seligman’s PERMA model, which includes the five elements of positive emotion, engagement, relationships, meaning, and accomplishment and was used to analyze the perspective of nurses’ professional self-concept. The inner circle introduces Bandura’s SCT, which was used to guide the construction of themes for the factors influencing nurses’ professional self-concept.

## Method

### Study design

This study employed a qualitative descriptive design to examine nurses’ perceptions of their professional self-concept and its influencing factors. This study adhered to the consolidated criteria for reporting in a qualitative research (COREQ) checklist [28] (Table S1).

### Study participants and sampling

This study was conducted in Guangdong Province, China, from January 23 to April 23, 2023. A total of 15 nurses, including three men and 12 women, were invited from six general tertiary-level hospitals in two cities (Shenzhen and Guangzhou) of Guangdong Province. The inclusion criteria were as follows: (1) age ≥ 18 years; (2) holding a nurse certificate; and (3) being a clinical nurse. The exclusion criteria are as follows: (1) being an intern or trainee nurse; (2) being a student or being on leave for one month or more; and (3) nurses who could not be contacted. Purposive and snowball sampling methods were used to invite participants to this study. Predetermined criteria were included according to the diversity of social demographic factors, such as working years, professional titles, and work departments, to obtain as rich data as far as possible. Participant selection was conducted by trained research team members. Invitations were distributed through WeChat, face-to-face, or by telephone to nurses. Snowball sampling was used to recruit more participants. The researchers explained the study’s purpose, precautions, and confidentiality principles to potential participants through WeChat or face-to-face conversations. Participation in the study was voluntary, and all participants provided written informed consent before participation.

Guest et al. [29] suggested that sample sizes for qualitative research are not predetermined; instead, sampling is considered to be saturated when no new data emerges, that is, when there are multiple repetitions of the data collected. They argued that for most research that aims to understand common perceptions and experiences, data saturation occurs after 12 interviews. As data saturation is a subjective judgment, we recruited additional interviewees after completing interviews with the initial 12 participants to ensure that our study achieved genuine data saturation, aiming for the highest level of comprehensiveness and quality. Thus, 17 participants were initially invited; however, two nurses declined to participate. Finally, 15 participants completed the interviews.

### Determining the interview outline

Based on the research purpose and a review of relevant literature, an initial interview outline was drafted. Two nurses were selected for the pre-interviews, and a final interview outline was developed after a group discussion. Two researchers (CYM and LQS) conducted a pilot test with two nurses separately, and neither of the nurses was invited to participate in the formal study. After the two pre-interviews, the formal interview outline was adjusted, improved, and finalized (The Interview Guide was developed for this study, see Table S2).

### Data collection

We conducted one-on-one, in-person, semi-structured interviews to gain insight into nurses’ perspectives and experiences. This approach fostered a comfortable environment for interviewees, encouraging them to share their personal opinions. Before the interviews, the researchers contacted the participants and clearly explained the research objectives, then arranged interview times and locations at the participant’s convenience. Locations free of distractions, such as conference rooms in the participants’ clinical department, their homes, or a quiet environment were selected.

During the interviews, the participants were informed of the necessity of recording the interview process. The purpose, process, and confidentiality principles of this study were explained again. Written informed consent was obtained. Each interview lasted 35–60 min. When necessary, the interviewer employed questioning, rhetorical questioning, and repetition techniques to confirm the participants’ responses and ensure clarity.

In the formal interview process, CYM conducted each interview while observing, listening to, and recording the interviewees’ expressions, voices, and intonation as well as clarifying and verifying uncertain information to improve the accuracy of the data. The interview guide consisted of open-ended questions that allowed participants to fully elucidate their viewpoints, perceptions, and experiences. At the beginning of each interview, the participants were asked to introduce themselves, including their department, working years, working position, and working experience, and then to explain their perceptions of “professional self-concept” and the “related influencing factors”. The researcher remained linguistically and nonjudgmental neutral in the interviews to observe and record. To express our gratitude, we also gave a gift card worth ¥100 to each participant who completed the interview.

### Data analysis

This study used qualitative thematic analysis to examine the nurses’ perceptions of the professional self-concept and its influencing factors. A thematic analysis method [30] was used to extract the factors affecting the professional self-concept among nurses, which consisted of the following six steps: (i) familiarization with the data, (ii) generating initial coding, (iii) searching for themes, (iv) reviewing the themes, (v) defining and naming, and (vi) generating reports. After obtaining consent, the recording was conducted using IFLYTEK recording equipment. Within 48 h after the interview, the researcher transcribed the recorded data into Microsoft Word by repeatedly listening to the recording content. The transcribed text was sorted, classified, coded, and analyzed using Nvivo 12.0 software.

### Details process of data handling and analysis

In the first step, CYM listened to the recorded data several times, read the transcribed text repeatedly, and noted any transcribed errors for timely correction. This process involved extracting relevant content from the transcript and adding the interviewer’s expressions in brackets based on the participants’ verbatim statements. Afterward, meaningful units were extracted from the transcribed text by condensing and summarizing recurring “words” or “sentences” as appropriate using the inductive analysis. XHL and CQL revised their interpretations of the findings if disagreements occurred during analysis. Once the open coding data were complete, CYM generated a list of categories and initial subthemes. Next, CYM, CQL, WYT and XHL reviewed all codes, categories, and initial subthemes that emerged from the transcripts. CYM and CQL then merged the items according to the categories developed from SCT theory using deductive analysis into relevant themes and subthemes. The combination of inductive and deductive approaches has been mentioned in previous studies [31]. As the team members held different views, the process was repeated until a consensus was reached. Finally, we used a table to report the entire thematic analysis findings process (Table S3), with quotes translated into English by CYM and XHL.

### Ethical approval

Before recruitment, we informed our study’s participants about the purpose, method, and content of the study. All participants were asked to sign a written consent form before the interview. In addition, the researchers used case numbers to anonymize the interview data and protect the interviewees’ privacy. All data used in the interviews were processed anonymously. Apart from de-identified records, no other relevant personal information was revealed. The study conformed to the Declaration of Helsinki. This study was approved by the Medical Ethics Committee of the Guangzhou Medical University (No. 202210003). This work is part of our overall research. Participation was entirely voluntary. Participants had full autonomy regarding whether to take part in this anonymous study.

### Trustworthiness and credibility

To improve the reliability of this study, we recruited participants with different characteristics, such as age, and nursing department, until we reached data saturation. After transcribing and collating the data, the researcher promptly checked and confirmed with the interviewees in cases of uncertainty in the information to ensure the authenticity and completeness of the data. The researcher kept detailed records of all the raw data and analyses and recalled and understood the interviews by listening to the recorded content several times and reviewing the translated text, thereby ensuring the credibility of the data. The detailed descriptions of each category enriched the interpretation of the data and enhanced transferability. In addition, themes and subthemes from the analyses were independently analyzed by two researchers to test the reliability of the results. We later contacted some participants, asked them to re-verify the content again, and asked them whether any corrections were needed. Moreover, if the participants asked us for the interview records, we provided them with the relevant details and content. However, owing to time constraints, we did not repeat the interviews.

### Results

**Fig. 2:**
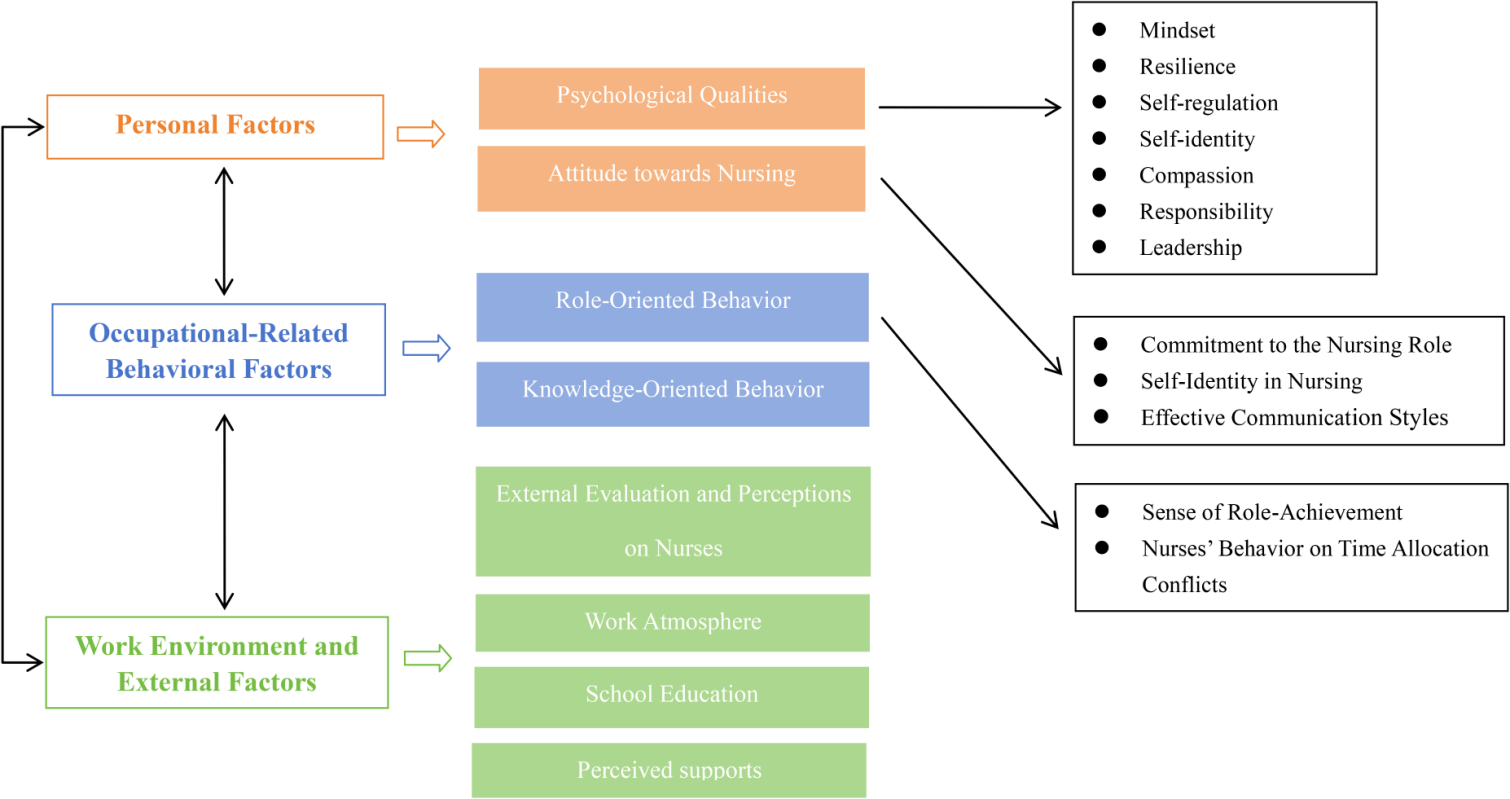
The summarizes of factors affecting nurses’ professional self-concept

No new topics were found after interviewing the 15 participants. The study sample was from six hospitals in two cities (Shenzhen and Guangzhou) of Guangdong Province, China. The participant’s characteristics are presented in Table 1. The interviews lasted 35–64 min and averaged 51 min long. The participants included 12 women and three men; Of them, 14 (93.34%) participants had a bachelor’s degree, and one had a postgraduate degree. The working hospitals included three hospitals each in Shenzhen and Guangzhou, Guangdong Province. Based on the SCT, we identified the following themes and subthemes by coding the interview transcripts: personal factors, occupational-related behavioral factors, work environment and external factors (Fig. 2). Data analysis generated 82 codes through the meaningful text, forming 18 initial subthemes in eight categories.

**Table 1:** Participant’s information

Serial number	Gender	Title	Working years	Education degree	Departments
A1	Male	Nurse	< 1 year	Bachelor’s	Internal medicine
A2	Female	Nurse	1–5 years	Bachelor’s	Emergency unit
A3	Male	Nurse	1–5 years	Bachelor’s	Surgical department
A4	Female	Nurse	1–5 years	Bachelor’s	Others
A5	Female	Nurse practitioner	1–5 years	Bachelor’s	Internal medicine
A6	Female	Nurse-in-charge	6–10 years	Bachelor’s	Surgical department
A7	Male	Nurse	1–5 years	Master’s	Intensive care unit
A8	Female	Nurse	< 1 year	Bachelor’s	Internal medicine
A9	Female	Nurse-in-charge	> 11 years	Bachelor’s	Internal medicine
A10	Female	Nurse-in-charge	> 11 years	Bachelor’s	Internal medicine
A11	Female	Nurse-in-charge	> 11 years	Bachelor’s	Psychiatry department
A12	Female	Nurse-in-charge	6–10 years	Bachelor’s	Surgical department
A13	Female	Nurse-in-charge	> 11 years	Bachelor’s	Obstetrics and gynecology
A14	Female	Nurse practitioner	6–10 years	Bachelor’s	Obstetrics and gynecology
A15	Female	Nurse practitioner	> 11 years	Bachelor’s	Obstetrics and gynecology

### Nurses’ perceptions of professional self-concept

Codes were classified into four descriptive themes: professional identity, competence, care, and knowledge (See Table S4). A total of 15 nurses were interviewed, and the majority (11/15) stated that this was the first time they had heard the term “self-concept”; however, few differences existed in their perceptions of professional self-concept. Most nurses felt that their self-concept reflected their role as health promoters, with a sense of identification with their role or profession. They stated that self-concept involves professional nursing skills, professional knowledge, humanistic care, and the influence of their thoughts and attitudes on their work behaviors. For instance, Participant A8 (nurse, woman, 23 years old) said, *“Being engaged in the nursing profession involves several aspects. First, it requires an understanding of one’s behavior in a professional context. Nurses need to be self-aware, knowing who they are in this role. They must decide how to act and regulate their behavior effectively. This process involves an ideological aspect as well, which is developing a self-perception and attitude that are appropriate for their professional responsibilities”.* In addition, some participants emphasized the importance of cultivating mental health literacy when mentioning this concept. *“First, we should develop a concept of self and focus on ourselves. Then, we should stay positive, and let that positivity influence our work. That’s also how you bring a positive influence to the patients” (A10, nurse in charge, woman, 33 years old)*.

## Factors influencing nurses’ professional self-concept

### Theme one: personal factors

#### Subtheme one: psychological qualities

In this study, several nurses considered psychological quality to be an important personal factor affecting their professional self-concept, particularly mindset, resilience, self-regulation, compassion, responsibility, and leadership.

##### Mindset

Given the challenging and stressful nature of nurses’ work environment, nurses with a positive mindset tend to be motivated and driven in their work; therefore, they are motivated in their professional development. One participant stated, *“Maintaining a positive and optimistic mindset is crucial for nurses to handle challenging things, such as emotional fluctuations when patients’ family members do not understand. Without it, nurses may risk facing increasing distress and potentially experiencing a sense of despair in their profession” (A14, Nurse practitioner, woman, 32 years old).*

##### Resilience

Resilience, which encompasses the ability to effectively control one’s emotions and maintain composure under perceived stress, adapt flexibly to the environment, and understand others, is key to an individual’s professional development. One participant stated, *“To smooth your career path in nursing, it’s vital to learn how to read situations and understand others’ perspectives. Avoid losing your temper whenever possible and maintain control over your emotions. I believe mastering this skill is crucial and will significantly benefit your professional life. These insights are based on my personal experiences and advice” (A12, woman, nurse-in-charge, 29 years old).*

##### Self-regulation

Effective self-regulation is necessary because individuals may experience setbacks at work. For example, one participant said, *“When dealing with psychiatric patients who express constant negativity, often due to resentment from necessary restraints upon admission, it’s vital to learn self-regulation to cope with their adverse emotions and behaviors” (A11, nurse practitioner, woman, 35 years old).*

##### Compassion

Compassion refers to putting oneself in the patient’s position. One participant reported, *“It is essential to remember that compassion and empathy are crucial, especially when interacting with patients. We should see them as more than just patients–they are pregnant women and babies. Being warm and caring towards people is a fundamental quality, regardless of your profession” (A15, nurse practitioner, woman, 45 years old).*

##### Responsibility

Responsibility is considered a fundamental component of prudence and mental well-being and constitutes an essential quality that nurses should possess. *“In our profession, where we handle life, being cautious and responsible is fundamental. In nursing, exercising discretion and possessing a strong sense of responsibility are basic requirements. If you’re accountable to your patients and dedicated to your profession, you’ll naturally be more meticulous, which ultimately benefits the patients” (A12).*

##### Leadership

Nurse leadership is fundamental to realizing a nurse’s values, and its development is essential for the successful practice of the nurse professionals. Although some nurses felt that leadership was more often found among senior nurses, one participant stated, *“While much of what I have mentioned may not seem directly related to authority or majesty, as you accumulate years of experience in your profession, it inherently represents a form of leadership”(A11).* This was equally important for younger nurses. Leadership can be a collection of personal qualities, intelligence, and character, as well as a drive and self-leadership to become a better and better version of oneself. One participant said, *“Been on the job for six months now, and I’m pretty much-handling things on my own. Got the hang of the basics, no need to bother them (either leaders or colleagues) for every little thing” (A8).*

#### Subtheme two: attitude towards the nursing profession

##### Commitment to the nursing role

Nurses’ commitment to their roles was reflected in their engagement. For instance, some respondents said that they were devoted to daily work, such as checking a patient’s information and asking for their name before giving an injection to ensure that the correct person is being treated. *“It has become a muscle memory; it cannot be erased, and I feel so involved that I forget that feeling of self (laughs)” (A3, nurse, man, 28 years old).* Some respondents even reported that they were always in an engaged state at work. *“From the beginning to the end of my work day, I am in a state of total mental tension. I would say that this is commitment” (A2, nurse, woman, 24 years old).*

##### Self-identity in nursing

Most nurses appreciated their profession and were willing to work in these roles. One participant said: *“Nurses are a profession that I feel has a sense of value and presence as well. I mean, I am proud to be doing this for a living”(A2). “People may think that nurses are only assistants to doctors, but in fact, doctors need to rely on us nurses instead…Nurses know better what patients need and the problems they need to solve” (A1, nurse, man, 24 years old).*

##### Effective communication styles

Effective communication styles could reflect nurses’ positive attitudes toward nursing care. One participant said, *“Effective communication is also important. Even if you excel in your professional skills and have a caring attitude, it will not work without good communication. Being outgoing and having a genuine connection with patients are equally necessary. Developing strong communication skills is a must”(A9, nurse in charge, woman, 33 years old).*

### Theme two: occupational-related behavioral factors

#### Subtheme one: role-oriented behavior

##### Sense of role-achievement

The source of nurses’ sense of achievement is reflected in the fact that their nursing work helps patients, producing not only a sense of individual value but also social value. *“I remember a particular incident with an overweight male patient who had difficult-to-locate veins. Other colleagues had tried and failed to draw his blood without causing him pain and bruising, leading to his dissatisfaction. However, during my night shift, I successfully drew his blood. He was amazed at how painless it was and expressed his surprise, saying,* *‘Is it done already? It didn’t hurt at all!’ (pop-eyed with excitement). He then praised me in front of my colleagues and leaders and during the clinical rounds. This incident boosted my confidence and pride in my skills, making me feel more assured in demonstrating my capabilities! (big smile and with a gleam in his eye)” (A3).*

##### Nurses’ behavior on time allocation conflicts

The time conflict involved in nursing tasks primarily includes nurses’ task allocation and learning and working time allocation. For instance, one participant said, *“When I’m eating and a new patient arrives, I face a dilemma: continue my meal with only half an hour left, or attend to the patient? It’s a conflict of interests. At such moments, it’s crucial to consider our role. As a nurse, during my 8-hour shift, I need to prioritize my professional responsibilities over personal needs. Wearing the nurse’s uniform means not always doing what I want; it’s about balancing conflicting interests while staying true to our nursing role” (A11).*

#### Subtheme two: knowledge-oriented behavior

Knowledge reserves are essential for nurses’ perceptions, clinical judgments, and decision-making, particularly for new nurses and nurses with little experience (e.g., less than ten years). For instance, one participant said: *“In clinical work, you often encounter things not taught in school or books. It can be really tough when patients ask about these areas, because you don’t always know the answers and you can’t just guess” (A8).*

In this study, several participants were fully aware of the importance of knowledge and referred to self-directed learning behaviors. *“I am not very familiar with some specialties of my department, such as Central Venous Catheter (CVC) and Extracorporeal Membrane Oxygenation (ECMO). Then, I might check the* *relevant operating guides* *to understand* *how it works” (A5, nurse, woman, 25 years old).*

### Theme three: work environment and external factors

#### Subtheme one: external evaluation and perceptions of nurses

Social stereotypes can significantly impact all aspects of life, particularly individuals’ confidence and motivation. For instance, when discussing the status of nurses, one participant said, *“When you say that you are a bachelor’s degree nurse, people are amazed. There are some (patients), of course, most (patients) nowadays probably do not have that point of view anymore, but there will still be a lot of (patients), some even who call you ‘waiter’ all the time…” (A10)*.

In addition, gender stereotypes exist about the role of nurses, in addition to stereotypes in terms of social hierarchy, as demonstrated by the fact that most people think of nurses as women. One participant said, *“While I am comfortable with my role as a male nurse within the hospital, I am aware that outside the hospital, the perceptions and comments of others can affect my confidence” (A3)*.

The value placed on nurses by outsiders (e.g., peers and the general public) motivates and encourages individuals to remain committed to this path. *“Since the epidemic, I feel the status of nursing staff has elevated. There’s a greater sense of respect for the nursing profession now compared to before” (A12)*.

#### Subtheme two: work atmosphere

Individuals are affected by an excellent work environment. *“The work environment where I am situated is relatively tidy, and the atmosphere among my colleagues is quite positive and energetic. It is an enthusiastic and forward-thinking team. Most people are eager to pursue their goals rather than thinking of nothing. I find this atmosphere to be quite favorable” (A12).*

#### Subtheme three: school education

Schooling plays a critical role in shaping nurses’ perceptions of professional self-concept. *“The school’s emphasis on humanistic qualities, theories, and education significantly shapes one’s growth. It is essential to clearly explain the duties and roles of nurses and how they contribute to society…” (A10).*

#### Subtheme four: perceived supports

First, the platform support impacts nurses’ professional development. One participant said, *“Whether a professional nurse or a consultant nurse, both need a suitable platform to grow and excel. Acknowledging that some platforms might not be as good may limit the diversity of a nurse’s insights into clinical conditions and problem-solving” (A13, Nurse in charge, woman, 45 years old).*

Second, the support of leaders is vital for nurses. One participant said, *“When I first joined, I was the only new one, without peers to confide in. My leader provided me with her own methods of psychological guidance, which helped me quickly adapt to the environment” (A14).*

Peer support can influence nurses’ motivation, thereby playing an integral role in their professional development. *“During the day shift, there’s always someone available to assist; you’re never left to handle everything on your own” (A2).*

Third, patient support is a source of motivation for nurses and a recognition of their professionalism. *“When I get that kind of patient affirmation, I actually feel that this career is very good ” (A8).*

Furthermore, nursing is a unique profession, and the attitude and care of family members also significantly affects the concentration and energy of individuals engaged in nursing work. *“My family members may say that they will take care of the child instead of going for a walk today” (A15).*

## Discussion

This study explored nurses’ perceptions of their professional self-concept and its influencing factors using the PERMA theory and SCT. Nurses regarded professional self-concept in four aspects: Identity, competence, care, and knowledge. Factors influencing professional self-concept were categorized into three themes: personal, occupational-related behavioral, work environment and external factors (Fig. 2). This study enriches the understanding of Chinese nurses’ perceptions of professional self-concept and its influencing factors. These findings can guide future interventions to develop and improve the nursing team and provide a foundation to further assist nursing managers in developing interventions and training to support and motivate nurses.

According to the participants, professional self-concept is multi-dimensional. The development of nurses’ professional self-concept was considered an important component of personal career development, as reflected in nurses’ goals in terms of professional competence and professional identity. This was in line with Ni et al.’s [32] conceptual understanding of career development. This further emphasizes the crucial role of nurses’ professional self-concept in their career development. In addition, participants highlighted the vital impact of mental health literacy (i.e., humanistic qualities and care) when discussing professional competencies. This concept has recently gained attention [33], particularly considering the stress of modern life. Mental health literacy is regarded as individuals’ knowledge and beliefs about recognizing, managing and preventing mental health problems [34, 35]. For nurses, improving mental health literacy not only means developing positive attitudes and practices, but it is also an important expansion of their professional self-concept. By developing mental health literacy, nurses can be helped to gain a deeper understanding of the complexity and importance of their own professional roles, thereby facilitating their professional growth and personal development [34]. Furthermore, the nurses participating in this study emphasized the prominent roles of caregiving and professional knowledge in their professional self-concept. This is linked to the pivotal role of nurses as healthcare providers in the medical and health fields, where they undertake responsibilities as caregivers and health educators. This alignment with prior research is consistent with delineating the dimensions of nurses’ professional self-concept [6], suggesting that nurses still have substantial room for growth in professional care and knowledge.

### Nurses’ mindsets, psychological qualities, and attitudes as an internal driver for the development of their professional self-concept

Regarding personal factors, we found that nurses’ mindsets and psychological qualities are a more significant part of the process of their professional self-concept development and career promotion. This was consistent with the findings of previous research [5, 7, 32]. According to Madrid et al., individuals’ positive or negative emotions at work may affect their self-perceptions and job satisfaction [36]. Nurses with a positive mindset have also been found to be able to work creatively. This may be because people with high levels of happiness accumulate more positive emotions, are satisfied with their lives, and can positively influence their organizational performance, which in turn positively affects the quality of care delivery [37]. Moreover, nurses with positive psychological qualities, such as resilience, and empathy, tend to maintain consistent positive expectations about future outcomes, which then leads to more positive outcomes that enhance their mental health, job satisfaction, professional self-concept [38–40], and career decision-making [41]. However, individual attributes take longer to develop and may be influenced by the environment, education, and experience [36]. Therefore, the joint efforts of nurses, families, and society, such as using a positive psychological intervention [42], are required to help nurses develop positive psychological and qualities to better promote the development of professional self-concept. For example, schools emphasize theoretical and humanistic qualities at the organizational level, including education and hospital management, whereas hospitals concentrate on operational and individual competencies. Teachers are key to shaping students’ qualities, values, and professional growth. Therefore, new teachers must possess a depth of knowledge and humanistic qualities to enrich students’ practical experiences and cultivate solid interpersonal abilities for effective, positive clinical adaptation [43].

Furthermore, nurses are the mainstay of clinical care, and their attitudes are key factors in shaping the overall quality of care. A positive, optimistic, and confident attitude toward life can lead to quality nursing care and inspire nurses to commit to their work [44]. Although the majority of the participants in this study indicated that they maintained a focused and devoted attitude towards their nursing work, some participants mentioned potentially negative attitudes owing to work pressure or for other reasons. A survey of 357 nurses in five hospitals in Ethiopia found that only 46.3% of nurses were optimistic about their careers [45]. This finding suggests that nurses should enhance their professional role clarity. In addition, a survey of 1,179 Austrian nurses found that they had moderate to positive attitudes towards caring for patients aged > 80 years [46]. However, previous research has also found that nurses’ attitudes towards others, such as caring for older people, are complex, with both positive and negative aspects [47, 48]. Thus, consistently positive attitudes towards nurses should be developed. Ethical training and continuous educational opportunities should also be provided [45].

Besides, previous studies have shown that nurses’ attitudes stem from their self-perception of the profession and that self-identification is an important part of this perception. Most of the nurses in this study still had a high level of acceptance of nursing as a profession and felt that it was a very rewarding job to have. This result was similar to that of previous studies in that those with high self-identity tended to have stronger self-confidence, had a clearer understanding of their abilities and values, were more likely to have stable and healthy relationships and were motivated to achieve their goals and aspirations. However, surveys conducted during the COVID-19 pandemic found that most nurses needed to promote their self-identity [49, 50]. For instance, Zhang et al. reported that, among 348 Chinese nurses, most reported that their professional self-identity was low or moderate [49].

In addition, some nurses in this study also reported that they needed to further improve their communication skills. To further enhance nurses’ professional self-concept, nurses’ psychological problems should be recognized early, and psychological intervention support and emotional management training should be provided. Moreover, relevant education and training, such as strengthening effective communication and interpersonal skills, should be provided, thereby inspiring nurses to be more passionate and committed to nursing and to maintain their long-term positive attitudes.

### Stimulating the autonomy of nurses’ occupational-related behavior is a synergistic force that improves their professional self-concept development

According to the SCT framework, an individual’s behavior plays a key role in their professional development. This study concluded that nurses’ occupation-related behaviors influenced their professional self-concept, with role-oriented behavior being the main aspect. This study examined nurses’ perceptions of job fulfillment and motivation, as well as their willingness to actively choose to take on the nursing role in the event of a conflict between family roles and time allocation. Nurses expressed that their motivation towards professional development was connected to the meaningful work they gained from their work, which is consistent with the results of previous studies [51]. Nurses’ intrinsic autonomy must be stimulated to develop and nurture this behavior. Studies have shown that individuals with a higher level of autonomy are more likely to take on responsibility and are more flexible, proactive, open to challenges, and adaptable to the content and demands of their work. Consequently, nursing managers can develop training programs to assist nurses in adapting to and developing their professional roles. In addition, regarding role conflict, some nurses indicated that they would be willing to meet patients’ needs over their own. However, choosing a role may be challenging owing to work-family conflict [52]. Thus, nurses require help to balance work and family life, including developing their coping strategies, organizational policies, and culture [53].

Moreover, nurses’ occupation-related behaviors are manifested as knowledge-oriented behaviors. In this study, they were manifested as nurses being aware of their own inadequacies and acting accordingly, such as seeking advice from others on job content or expertise. Previous studies have also found that nurses’ knowledge and skills in certain specialized areas require improvements [54], and that nurses need help to better cope with the challenges they face through more educational and clinical practice opportunities [52]. Furthermore, previous research has emphasized the importance of knowledge in providing adequate care, including health promotion and disease prevention. Nurses also need to be able to locate the required knowledge and its sources. Nurses should not limit their knowledge to textbooks; they should also know how to apply it and translate it into action to develop their competencies [55, 56].

Accordingly, nurses’ occupational-related behaviors can be promoted through the development of their professional competencies, such as the development of cyclical work plans, setting specific self-improvement goals, and proactive pursuit of various learning opportunities.

### Creating a favorable environment that enhances nurses’ perceived support and sense of belonging is an external motivation to enhance their professional self-concept

In this study, a favorable environment and external related factors, including the work environment in which an individual is placed, others’ perceptions, atmosphere, and perceived support, may serve as extrinsic motivations for an individual’s career development and professional self-concept.

First, others’ perceptions of the nurses’ roles are crucial. Some nurses felt that others’ perceptions of nursing roles could be considered one of the factors influencing their professional self-concept. Kallio et al. [51] found that these perceptions significantly affected nurses’ physical and mental health and influenced their job retention. Negative perceptions of nurses’ roles by others may lead to role conflicts. The public’s perception of the role of nurses has changed from the original daily auxiliary work, such as giving injections and medicines. However, establishing and maintaining a good image among nurses is a long-term process that requires joint efforts of the nursing community and the outside community.

Second, a favorable working environment, including providing a group of nurses with an adequate sense of support and belonging, is an important component of their psychological needs for self-actualization [57]. A positive working atmosphere, with harmonious interpersonal relationships and mutual trust among members, enhances team cohesion and contributes to team development. In line with the findings of Drott et al. [58], we found that the interactive relationships between leaders and their subordinates as well as employees’ supportive aspects can affect individuals’ development. A qualified leader can think differently and provide help and guidance to subordinates, thus motivating and driving the entire nursing team in the department [59].

Additionally, schools emphasize theoretical and humanistic qualities at the organizational level, including education and hospital management, whereas hospitals concentrate on operational and individual competencies. School and clinical education play important roles in the early formation and long-term development of nurses’ professional self-concept, which is in line with previous studies [60, 61]. Teachers play a central role in shaping student qualities and professional values, imparting education, and influencing their clinical adjustment and professional development. Hence, nursing teachers should possess a solid foundation of knowledge and humanistic qualities to influence students’ clinical adjustment and professional development effectively. Furthermore, they should enhance nursing students’ practical experiences and cultivate solid interpersonal skills to positively impact their clinical adaptation [43].

In addition, favorable social support contributes to nurses’ career development. This study found that nurses who perceived themselves to have greater support from society, organizations, and peers could face work stress and challenges using positive strategies. This was consistent with the findings of Liu et al. [62]. Moreover, Cao et al. [63] suggested that a positive work environment motivates individuals to work harder to achieve their career development. For instance, a positive coworker relationship could promote career development by allowing individuals to feel safe in their group, trust others, and learn from each other [64]. Moreover, in a leadership relationship, Kallio et al. [51] reported that support for nurses’ career development by nursing managers is very important, as nurses’ perceived limitations in their career development are one of the reasons that lead them to choose to leave the nursing profession. Thus, nursing managers should be able to provide nurses with career planning assistance, targeted motivation, and encouragement to participate in various competitions and training opportunities. This would help them recognize their strengths and develop motivation to grow in their nursing careers.

### Limitations

This study provides a rich understanding of Chinese nurses’ perceptions of their professional self-concept and the influencing factors. These findings further enrich the theoretical framework of professional self-concept. These factors may be beneficial for advancing nurses’ career development. However, this study had some limitations. First, the interviewer attempted to recall and document the interviewees’ facial expressions during the one-on-one interviews. However, capturing and recording all the participant’s facial expressions and movements was difficult, resulting in some potential oversights. Second, some nurses declined to participate because of time constraints associated with face-to-face communication. The gender bias in the sample, which was predominantly made up of women and had a small sample size, caused the research methodology and purposeful sampling, to restrict the generalization of the results to be broader nursing population. Third, the results of this study may be biased towards exploring the factors influencing nurses’ professional self-concept, and personal bias associated with the influence of the environment, such as others’ opinions on nurses, may be present. Interviewers’ interpretation and construction may have affected the data collection and analysis, and some of the content may not have been explored in-depth. Third, quotes were translated from Chinese into English, and the meanings of the translated quotes may differ slightly from the original meanings in Chinese. Moreover, although the results of this study shed light on Chinese nurses’ perceptions of the components and factors of professional self-concept, this study did not describe the interrelations between the components and factors. Therefore, the findings of this study should be interpreted carefully.

## Conclusions

This study provides new insights into nurses’ perceptions of professional self-concept and its influencing factors based on PERMA theory and SCT. First, nurses emphasized professional identity, competence, care, and knowledge as the primary components of professional self-concept, which indicates that nursing managers should pay closer attention to these areas. Second, according to nurses, three themes and eight subthemes in personal, behavioral, and external aspects that affect nurses’ professional self-concept have been identified. Adopting differing positive methods in accordance with these themes and factors, such as promoting nurses’ positive qualities, attitudes, and behaviors and establishing a good support system, can be used as a foundation to enhance nurses’ professional self-concept and development. Additionally, it needs to be highlighted that enhancing nurses’ professional self-concept requires not only the nurses themselves, but also the joint efforts of patients, their families, the healthcare system, and society as a whole.

### Electronic supplementary material

Below is the link to the electronic supplementary material.

Supplementary Material 1

## Data Availability

All the raw data (including participants’ voice files and the texts of the interviews) will be confidential and will not be able to share publicly. However, the codes that emerged during the current study are available from the corresponding author upon reasonable request.
