# Supplementary material for: Nurses’ perspectives on professional self-concept and its influencing factors: A qualitative study
Source: BMC Nurs. 2024 Apr 9;23:237. doi: 10.1186/s12912-024-01834-y (PMC11003037; doi:10.1186/s12912-024-01834-y)
Supplement: Supplementary file 1 — Supplementary Material 1 [file 12912_2024_1834_MOESM1_ESM.doc]

**Table S1. COREQ (****Consolidated criteria for Reporting Qualitative research) Checklist**

| **No. Item** | **Guide questions/description** | **Reported on Page #** |
| --- | --- | --- |
| **Domain 1: Research team and reflexivity** |  |  |
| *Personal Characteristics* |  |  |
| 1. Interviewer/facilitator | Which author/s conducted the interview or focus group? | First author  Page 10 |
| 2. Credentials | What were the researcher’s credentials? E.g. PhD, MD | See related file |
| 3. Occupation | What was their occupation at the time of the study? | See related file |
| 4. Gender | Was the researcher male or female? | See related file |
| 5. Experience and training | What experience or training did the researcher have? | See related file |
| *Relationship with participants* |  |  |
| 6. Relationship established | Was a relationship established prior to study commencement? | Yes, page 8 |
| 7. Participant knowledge of the interviewer | What did the participants know about the researcher? e.g. personal goals, reasons for doing the research | Page 9 |
| 8. Interviewer characteristics | What characteristics were reported about the interviewer/facilitator? e.g. Bias, assumptions, reasons and interests in the research topic | Page 9 |

| **Domain 2: study design** |  |  |
| --- | --- | --- |
| *Theoretical framework* |  |  |
| 9. Methodological orientation and Theory | What methodological orientation was stated to underpin the study? e.g. grounded theory, discourse analysis, ethnography, phenomenology, content analysis | Page 10-11 |
| *Participant selection* |  |  |
| 10. Sampling | How were participants selected? e.g. purposive, convenience, consecutive, snowball | Page 8 |
| 11. Method of approach | How were participants approached? e.g. face-to-face, telephone, mail, email | Page 8 |
| 12. Sample size | How many participants were in the study? | Page 8-9 |
| 13. Non-participation | How many people refused to participate or dropped out? Reasons? | Page 8-9 |
| *Setting* |  |  |
| 14. Setting of data collection | Where was the data collected? e.g. home, clinic, workplace | Page 5 |
| 15. Presence of non-participants | Was anyone else present besides the participants and researchers? | No. This is a one-on-one interview.  Page 9 |
| 16. Description of sample | What are the important characteristics of the sample? e.g. demographic data, date | Table 1 |
| *Data collection* |  |  |
| 17. Interview guide | Were questions, prompts, guides provided by the authors? Was it pilot tested? | Yes, Table S2, Page 9 |
| 18. Repeat interviews | Were repeat interviews carried out? If yes, how many? | No, inferred on page 9 |
| 19. Audio/visual recording | Did the research use audio or visual recording to collect the data? | Use the recording equipment, Page 10 |
| 20. Field notes | Were ﬁeld notes made during and/or after the interview or focus group? | No, not applicable |
| 21. Duration | What was the duration of the interviews or focus group? | Page 10 |
| 22. Data saturation | Was data saturation discussed? | Page 8 |
| 23. Transcripts returned | Were transcripts returned to participants for comment and/or correction? | Page 12 |
| **Domain 3: analysis and ﬁndings** |  |  |
| *Data analysis* |  |  |
| 24. Number of data coders | How many data coders coded the data? | Page 13 |
| 25. Description of the coding tree | Did authors provide a description of the coding tree? | Yes, Table 2 |
| 26. Derivation of themes | Were themes identiﬁed in advance or derived from the data? | Identified in advance, Table 2 |
| 27. Software | What software, if applicable, was used to manage the data? | Page 11 |
| 28. Participant checking | Did participants provide feedback on the ﬁndings? | Page 12 |
| *Reporting* |  |  |
| 29. Quotations presented | Were participant quotations presented to illustrate the themes/ﬁndings? Was each quotation identiﬁed? e.g. participant number | Yes, Page 14-22 |
| 30. Data and ﬁndings consistent | Was there consistency between the data presented and the ﬁndings? | Yes, there was.  Page 17 |
| 31. Clarity of major themes | Were major themes clearly presented in the ﬁndings? | Yes. they were.  Table 2 |
| 32. Clarity of minor themes | Is there a description of diverse cases or discussion of minor themes? | Yes. Discussion of major and minor themes  Table S3 |

Developed from:

Tong A, Sainsbury P, Craig J. Consolidated criteria for reporting qualitative research (COREQ): a 32-item checklist for interviews and focus groups. *International Journal for Quality in Health Care*. 2007. Volume 19, Number 6: pp. 349 – 357

**Table S2. Interview Guide**

| No. | Questions |
| --- | --- |
| Beginning | [Greetings] We are delighted to see you participate in this interview. |
|  | Ask the interviewee to briefly introduce themselves. |
| Q1 | What is your understanding and opinion of the term “nurses’ professional self-concept”? Why? |
| Q2 | What factors do you believe are related to nurses’ professional self-concept? Why? |
| Q3 | How do you think and experience your role as a nurse? |
| Q4 | What characteristics and abilities do you think nurses should have? |
| Q5 | What is your perspective on the relationship between nurses and others? Why? |
| Q6 | What makes you feel more engaged in your work? |
| Q7 | What makes you feel accomplished, proud, or valuable in your work? |
| Q8 | What advice do you have for successfully qualifying as a nurse? |
| Additions | Is there something else you would like to add? |

| **Table S3. The thematic analysis process of influencing factors of nurses’ professional self-concept** | | | | | |
| --- | --- | --- | --- | --- | --- |
| Themes | Subthemes | Initial subthemes | Categories | Codes (examples) | Meaningfully sentence (examples) |
| Personal factors | Psychological qualities | Mindset | Positive mindset | A positive and optimistic mindset is needed in challenging situations | *Maintaining a positive and optimistic mindset is crucial for nurses to handle challenging things, such as emotional fluctuations when patients' family members do not understand. Without it, nurses may risk facing increasing distress and potentially experiencing a sense of despair in the nursing profession (A14).* |
|  |  | Resilience | Keep calm, and resilience | Learn to read people’s expressions and words, control your emotions, and refrain from easily losing your temper | *To smooth your career path in nursing, it’s vital to learn how to read situations and understand others’ perspectives. Avoid losing your temper whenever possible and maintain control over your emotions. I believe mastering this skill is crucial and will significantly benefit your professional life (A12).* |
|  |  |  | Acceptance of criticism and recommendations | Requires a certain level of psychological resilience | *In clinical operations, it’s unrealistic to expect perfection. Thus, when receiving instruction or criticism, it’s crucial to understand it’s about the task, not a personal attack, and to maintain mental resilience (A10).* |
|  |  | Self-Regulation | Acceptance of negative emotions and the ability to self-regulation | Properly and reasonably alleviate negative emotions, have the ability to self-regulation | *When dealing with psychiatric patients who express constant negativity, often due to resentment from necessary restraints upon admission, it’s vital to learn self-regulation to cope with their adverse emotions and behaviors (A11).* |
|  |  | Compassion | Patience, and compassion | Compassionate and patient-centered | *He (the patient) may ask a question hundreds of times daily, and you must keep answering his questions. You must tolerate some patients whose behavior is very chaotic, so you have to be loving, which is very important (A11).* |
|  |  | Responsibility | Possess a sense of responsibility | Nurses’ roles require a spirit of prudence, including responsibility, and professional dedication | *In our profession, where we handle life, being cautious and responsible is fundamental. In nursing, exercising discretion and possessing a strong sense of responsibility are basic requirements. If you’re accountable to your patients and dedicated to your profession, you’ll naturally be more meticulous, which ultimately benefits the patients (A12).* |
| Table S3. *(Continued)* | | | | | |
| Themes | Subthemes | Initial subthemes | Categories | Codes (examples) | Meaningfully sentence (examples) |
| Personal factors | Psychological qualities | Leadership | Self-leadership | Have the ability to handle things independently | *Been on the job for six months now, and I’m pretty much handling things on my own. Got the hang of the basics, no need to bother them (either leaders or colleagues) for every little thing (A8).* |
|  |  |  | Superior Leadership | Hold a certain level of authority | *Relatively speaking, I will know a little bit more than they do. After all, I will have worked in the clinic for more time than they do, and I still have a certain degree of authority at times (A12).* |
|  | Attitude towards the nursing profession | Self-identity in nursing | Nurses play an essential role | Nurses are not just doctors’ assistants; they are indispensable to clinical practice | *People may think that nurses are only assistants to doctors, but in fact, doctors need to rely on us nurses instead...Nurses know better what patients need and the problems they need to solve (A1).* |
|  |  | Commitment to the nursing role | Engagement | Dedication to work | *When performing any procedure, I am always fully engaged because I understand that a lack of involvement could lead to serious errors (A15).* |
|  |  | Effective communication styles | Communication with others | Effective communication skills are needed | *Effective communication is also important. Even if you excel in your professional skills and have a caring attitude, it will not work without good communication. Being outgoing and having a genuine connection with patients are equally necessary. Developing strong communication skills is a must (A9).* |
| Table S3. *(Continued)* | | | | | |
| Themes | Subthemes | Initial subthemes | Categories | Codes (examples) | Meaningfully sentence (examples) |
| Occupational behavior Factors | Role-oriented behavior | Sense of role-achievement | Professional satisfaction and fulfillment | Sense of fulfillment from work | *My experience so far has been very satisfying. Being able to help others is a joy. I remember a particular incident with an overweight male patient who had difficult-to-locate veins. Other colleagues had tried and failed to draw his blood without causing him pain and bruising, leading to his dissatisfaction. However, during my night shift, I successfully drew his blood. He was amazed at how painless it was and expressed his surprise, saying, ‘Is it done already? It didn’t hurt at all!’ (pop-eyed with excitement). He then praised me in front of my colleagues and leaders and when on the clinical rounds. This incident boosted my confidence and pride in my skills, making me feel more assured in demonstrating my capabilities**! (a gleam in his eye) (A3).* |
|  |  | Nurse’s behavior on time allocation conflicts | Inadequate time for mission | Conflict between family and work | *There are so many things to learn, I feel that I don’t have enough time... In fact, a nurse doesn't only have a job but also has to study, attend training, and have her own family (A4).* |
|  |  |  |  | The conflict between self-rest and work | *When I’m eating and a new patient arrives, I face a dilemma: continue my meal with only half an hour left, or attend to the patient? It’s a conflict of interests. At such moments, it’s crucial to consider our role. As a nurse, during my 8-hour shift, I need to prioritize my professional responsibilities over personal needs. Wearing the nurse’s uniform means not always doing what I want; it’s about balancing conflicting interests while staying true to our nursing role (A11).* |
| Table S3. *(Continued)* | | | | | |
| Themes | Subthemes | Initial subthemes | Categories | Codes (examples) | Meaningfully sentence (examples) |
| Occupational behavior Factors | Knowledge-oriented behavior | Self-perceived behaviors | Rationally recognize difficulties | Perceive the importance of learning knowledge | *While working in the inpatient unit, I focused primarily on routine tasks and neglected the opportunity to enhance my professional knowledge and skills. Consequently, when I transitioned to the emergency department, I realized I lacked familiarity with certain professional knowledge, such as the underlying theory (A2).* |
|  |  | Professional knowledge | Professional Knowledge | Importance of professional knowledge to nurses’ profession | *In clinical work, you often encounter things not taught in school or books. It can be really tough when patients ask about these areas because you don't always know the answers and you can't just guess (A8).* |
|  |  | Autonomous behaviors | Active learning and thinking | Great experience with other staff | *I often reflect on what I can learn from my colleagues, particularly their positive work habits, and strive to incorporate these into my own practice in my work (A2).* |
|  |  |  | Expectations for learning | The desire to learn knowledge | *For the future, I aspire to further my education. My goal is to enhance my academic qualifications through work, which would enable me to gain more exposure, pursue advanced studies, and learn about practices in other hospitals. I’m particularly interested in expanding my knowledge in specialized areas of our field (A5).* |
| Work Environment and External Factors | External evaluation and perceptions of nurses | External perceptions and opinions on the nurses’ role | The public’s perception of nurses has shifted | The status of nurses has improved | *Since the pandemic, I feel the status of nursing staff has elevated. There’s a greater sense of respect for the nursing profession now compared to before (A12).* |
| Table S3. *(Continued)* | | | | | |
| Themes | Subthemes | Initial subthemes | Categories | Codes (examples) | Meaningfully sentence (examples) |
| Work Environment and External Factors | Work atmosphere | Work atmosphere | Positive working environment and atmosphere | The working environment is clean, and the team members are full of energy and enthusiasm | *The work environment where I am situated is relatively tidy, and the atmosphere among my colleagues is quite positive and energetic. It is an enthusiastic and forward-thinking team. Most people are eager to pursue their goals rather than thinking of nothing. I find this atmosphere to be quite favorable (A12).* |
|  | School and clinical education | School education | School education is essential is professional development | School shapes nurses' roles and their professional perspectives | *The school’s emphasis on humanistic qualities, theories, and education significantly shapes one’s growth. It is essential to clearly explain the duties and roles of nurses and how they contribute to society. This encompasses the unique presence or ‘angelic’ aspect we represent. The school is instrumental in developing a nurse's professional perspective (A10).* |
|  | Perceived supports | Perceived supports | Nurses need overall supports | Support from government | *The government has certain support for nurses in the professional title evaluation or employment scope...**(A13).* |
|  |  |  |  | Support from hospital platform opportunities | *Need hospitals to give a certain platform to achieve their own...(A14).* |
|  |  |  |  | Support from leaders | *When I first joined, I was the only new one, without peers to confide in. My leader provided me with her own methods of psychological guidance, which helped me quickly adapt to the environment (A14).* |
|  |  |  |  | Support form patients | *When I get that kind of patient affirmation, I actually feel that this career is very good (A8).* |
|  |  |  |  | Support from peers | *During the day shift, there’s always someone available to assist; you’re never left to handle everything on your own (A2).* |
|  |  |  |  | Support from family | *If the family can not give some support, the nurse may find it challenging to fully concentrate on the work (A11).* |

**Table S4. The concept of nurses****’ perceptions on professional self-concept**

| Theme | Categories | Codes | Meaningfully sentence (example) |
| --- | --- | --- | --- |
| Professional identity | Self-identity | Individual self-awareness and self-evaluation | *Self-concept should encompass an awareness of oneself, encompassing how one assesses and understands their own being (A9, Head nurse, Woman, 37 years old).* |
| Competence | Professional skills, communication, management, and humanistic quality | Related to self, self-profession, professional values, skills, or communication, management, and humanistic quality | *Initially, from a literal perspective, ‘self’ primarily refers to one’s profession, which is certainly tied to one’s personal expertise. This could be an aspect of self-concept related to one’s professional values, encompassing evaluations of potential skills, communication abilities, aspects of management, and humanistic quality (A6, Nurse, Woman, 29 years old).* |
| Care | Caring | Nursing is not just about giving injections and medicine, but also about providing person-centered care | *Before I became involved in this field, my understanding of the profession was limited to administering injections and dispensing medications. However, once I actually entered the field, I realized there’s a wealth of knowledge to be mastered. It's crucial for nurses to understand the patient’s medical conditions and the progression of their illnesses, as well as the psychological changes they undergo. Additionally, providing humane care is of utmost importance (A3, Nurse, Man, 26 years old).* |
| Knowledge | Knowledge | It refers to professional knowledge and the degree of mastery of one’s own professional knowledge | *This refers to my expertise in my field, which I believe I have a firm grasp on. I can effectively manage and control this knowledge area. In other words, I consider myself a professional, particularly in nursing. I feel this concept revolves around professionalism. Speaking of the self, I believe that I am in command of this professional knowledge; I am the one who steers its direction (A15, Nurse Supervisor, Woman, 45 years old).* |
